# Intermittent Information-Driven Multi-Agent Area-Restricted Search

**DOI:** 10.3390/e22060635

**Published:** 2020-06-08

**Authors:** Branko Ristic, Alex Skvortsov

**Affiliations:** 1 School of Engineering, RMIT University, Melbourne, VIC 3000, Australia; 2 Maritime Division, Defence Science and Technology Group, Fishermans Bend, VIC 3207, Australia; alex.skvortsov@dst.defence.gov.au

**Keywords:** autonomous search, infotaxis, multi-agent system, decentralised control

## Abstract

The problem is a two-dimensional area-restricted search for a target using a coordinated team of autonomous mobile sensing platforms (agents). Sensing is characterised by a range-dependent probability of detection, with a non-zero probability of false alarms. The context is underwater surveillance using a swarm of amphibious drones equipped with active sonars. The paper develops an intermittent information-driven search strategy, which alternates between two phases: the fast and non-receptive displacement phase (called the ballistic phase) with a slow displacement and sensing phase (called the diffusive phase). The proposed multi-agent search strategy is carried out in a decentralised manner, which means that all computations (estimation and motion control) are done locally. Coordination of agents is achieved by exchanging the data with the neighbours only, in a manner which does not require global knowledge of the communication network topology.

## 1. Introduction

Searching strategies for finding targets using appropriate sensing modalities are of great importance in many aspects of life, from national security [1,2], rescue and recovery missions [3,4], to biological applications [5,6,7]. A taxonomy of search problems is proposed in [8]. We focus on *probabilistic search*, where the objective is to localise the target in the shortest time (on average), for a given search volume.

The earliest theoretical studies of search strategies [9,10] were based on systematic search along a predetermined (deterministic) path, such as the parallel sweep or the Archimedean spiral [3,11]. The search patterns of animals, on the contrary, are random rather than deterministic. An explanation for this phenomenon is that an event, such as a detection (false or true), changes the strategy and hence the behaviour of the searcher. Subsequent changes of strategy manifest themselves as a random-like motion pattern. Most of the current research into search strategies is towards the mathematical modelling and explanation of random search patterns [2,12,13].

By studying the GPS data of albatrosses, it was discovered that search patterns of these birds consist of the segments whose lengths are random draws from the Pareto–Lévy distribution [6]. This discovery led to several papers demonstrating that the so-called Lévy walks/flight are the optimal search strategy for foraging animals (deer, bees, etc), resulting in fractal geometries of search paths. An alternative to Lévy strategies is the *intermittent search*: a combination of a fast and non-receptive displacement phase (long jumps within the search domain, with no sensing) with a slow search phase characterised by sensing and reaction [14]. Bénichou et al. provided both a theoretical study and experimental data verification of intermittent search [13,15]. In their terminology, the fast relocation phase is referred to as the *ballistic* flight with constant velocity and random direction. The slow sensing/detection phase is modelled as either a motionless wait or a *diffusive displacement*. Bénichou et al. studied intermittent search without taking into account the information gathered by sensing during the search, so the searcher could revisit a same location multiple times; this leads to apparent redundancy in the search process. To overcome this shortcoming, Vergassola et al. [16] proposed an information driven search strategy (referred as infotaxis), which selects the motion option that maximises the expected rate of the information gain. Information driven search by infotaxis made a profound impact on the research community (for a recent review, see [2]). Vergassola et al. considered information driven search only in the slow sensing/detection phase and for a single searching agent. Multi-agent infotaxis have subsequently been proposed in [17,18,19,20,21].

In this paper, we propose a fully decentralised intermittent information-driven search by a coordinated team of autonomous agents. Each searching agent is equipped with a sensor, characterised by unreliable detection, in the sense that the probability of correct detection depends on the distance to the target, while the probability of false detection is non-zero. Displacement decisions for each agent (i.e., where to move next), both in the ballistic phase and in the diffusive displacement phase, are based on maximisation of the expected information gain. Each searching agent performs the computations (estimation and motion control) locally and independently of other agents. Group coordination, for the sake of achieving the (common) task mission, is carried out via consensus [22], by exchanging the data only with neighbours, in a manner which does not require global knowledge of the communication network topology. The proposed approach is therefore scalable, in the sense that the complexities for sensing, communication and computing per agent are independent of the network and agent formation size. In addition, because all sensor platforms are treated equally (no leader–follower hierarchy), this approach is robust to the failure of any of the searching agents. The only requirement for avoiding the break-up of the multi-agent formation is that the graph of its communication network, during the search, is connected at all times.

## 2. Problem Formulation

For convenience, and without loss of generality, let us consider a search area A in the shape of a square with sides of length *b*. The area is discretised into an upright square lattice of the unit mesh size, thus consisting of $N=b^{2}\gg 1$ cells. The grid is specified as $G=\{\left(x_{n},y_{n}\right);n=1,\ldots ,N\}$, where $\left(x_{n},y_{n}\right)$ are the Cartesian coordinates of a centre of *n*th cell.

The team of searching agents consists of S≥1 members. Let the searching agent s∈{1,2,…,S} at discrete-time *k* be located in the cell $l_{k}^{s}\in G$. If the agent is in the sensing mode, it collects at time *k* a set of detections $Z_{k}^{s}=\left\{z_{k,1}^{s},\ldots ,z_{k,|Z_{k}^{s}|}^{s}\right\}$. Each detection can originate from the true target or be a false alarm. A vector $z\in Z_{k}^{s}$ consists of a range and azimuth measurement of the perceived target, relative to agent position $l_{k}^{s}$. Thus, if the true target is in the grid cell x∈G, its corresponding measurement is a Gaussian random vector z|x∼N(h(x),R), where

(1) $$h\left(x\right)=\left[\begin{array}{c} ∥l_{k}^{s}-x∥\\ ∠(l_{k}^{s},x) \end{array}\right],$$

∥·∥ is Euclidean distance, ∠(u) is the angle between u and the *y*-axis of the references coordinate system and R is the measurement covariance matrix. The probability of detection $P_{d}$ is a function of the distance between the agent and the target. The probability of false alarm $P_{fa}$ is constant within the sensing area around the agent (and zero otherwise). For example, let the probability of detection be adopted as $P_{d}=\exp\left(-r/a\right)$, where *r* is the distance between the target and the agent and a=const is a sensor characteristic. Assuming $360^{{}^{\circ}}$ sensor coverage, the sensing area can be defined as the circular area around the sensor position, with a radius 3a (in this area $P_{d}>0.05$).

**Figure 1:**
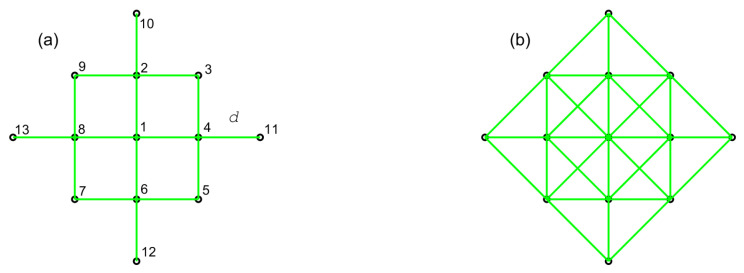
A team of 13 searching agents connected with different communication networks: (**a**) $R_{\max}=1.1d$; and (**b**) $R_{\max}=2d$. *d* is the smallest distance between the agents. Both communication graphs are connected.

Searching agents move in formation. Each agent knows its relative coordinates within the formation (for example, the offset from the centroid), however, it does not have to know the topology of the formation or its size. Communication between two agents in the formation can be established only if their mutual distance is smaller than some $R_{\max}$. Motion is subjected to small perturbation errors, meaning that an agent whose destination during the displacement is a cell l∈G, may end up in a cell adjacent to it. These motion errors will cause the network topology to vary with time. For simplicity, we assume that communication links, when established, are error-free. Figure 1 shows a formation of 13 searching agents with two different communication graphs for two values of $R_{\max}$. Green lines indicate the existence of a communication link between two nodes (agents) in the graph.

The problem for the team of agents is to coordinate the search and find the target in the shortest possible time, using the information-driven intermittent search strategy. The described problem can be applied in the context of a search for a submarine, using a swarm of amphibious drones, each equipped with an active sonar system and a wireless communication device. When a drone floats on the sea surface, it turns on its sonar to collect detections of underwater targets.

## 3. Decentralised Estimation: The Probability of Occupancy Map

Let us denote the complete set of measurement data from all *S* agents at time *k* as $D_{k}\equiv {\left\{\left(Z_{k}^{s},l_{k}^{s}\right)\right\}}_{s=1,\ldots ,S}$, where $Z_{k}^{s}$ and $l_{k}^{s}$ represent the measurement set and the location of *s*th agent, respectively, at time *k*.

The current knowledge about the target location within the search area A is represented by the *probability of occupancy map* (POM). This is a map in which each pixel corresponds to a cell of the grid G and represents the posterior probability of target presence in the cell. For cell *n* and agent s∈{1,…,S}, this posterior probability at time *k* is expressed as $p_{k,n}^{s}=Pr\left\{\delta_{n}=1|D_{1:k}\right\}$, where $D_{1:k}\equiv D_{1},D_{2},\ldots ,D_{k}$, and $\delta_{n}\in \left\{0,1\right\}$ is a Bernoulli random variable representing the event that target is located in cell *n*. The POM is then a collection $P_{k}^{s}=\left\{p_{k,n}^{s};n=1,\ldots ,N\right\}$.

The POM is updated sequentially using the Bayes rule. Given the POM at the previous time, that is $P_{k-1}^{s}$, and the measurement data at time *k* from agent t∈{1,…,S}, that is $\left(Z_{k}^{t},l_{k}^{t}\right)$, the probability of occupancy in the *n*th cell is updated as [23]

(2) $$p_{k,n}^{s}=\frac{\left(1-P_{d}^{t,n}\right)p_{k-1,m}^{s}}{\left(1-P_{d}^{t,n}\right)p_{k-1,n}+\left(1-P_{fa}^{t,n}\right)\left(1-p_{k-1,n}^{s}\right)}$$

if none of the detections in $Z_{k}^{t}$ falls into the *n*th cell. The term $P_{d}^{t,n}$ in Equation (2) represents the probability of detection in the *n*th cell given that the *t*th agent is located at $l_{k}^{t}\in G$. A similar explanation applies to the probability of false alarm $P_{fa}^{t,n}$. If a detection from $Z_{k}^{t}$ falls in the *n*th cell, the update equation for the POM of agent *s* is:

(3) $$p_{k,n}^{s}=\frac{P_{d}^{t,n}p_{k-1,n}^{s}}{P_{d}^{t,n}p_{k-1,n}^{s}+P_{fa}^{t,n}\left(1-p_{k-1,n}^{s}\right)}.$$

Initially, before any sensing information is received, that is at k=0, the POM of each agent is set to $p_{0,n}^{s}=\frac{1}{2}$, for all n=1,…,N and s=1,…,S. This POM corresponds to the state of complete ignorance.

Each agent in the team updates sequentially its local POM using its local measurements and those measurements it receives from other agents in the team (ideally, using the complete set of data $D_{k}$). We adopt the dissemination based decentralised architecture [24] for this purpose, where the entire $D_{k}$ is exchanged via an iterative scheme over the communication network. In the first iteration, agent *s* broadcasts its data-pair $\left(Z_{k}^{s},l_{k}^{s}\right)$ to its neighbours and receives from them their respective data-pairs. In the second, third and all subsequent iterations, agent *s* broadcasts its newly acquired data-pairs to its neighbours and accepts from them only the data-pairs that agent *s* has not seen before (i.e., newly acquired). Providing that the communication graph is connected, after a sufficient number of iterations (which depends on the topology and the size of the graph), a complete list of measurement data pairs from all agents in the formation (i.e., $D_{k})$, will be available to each agent for updating its local POM.

**Figure 2:**
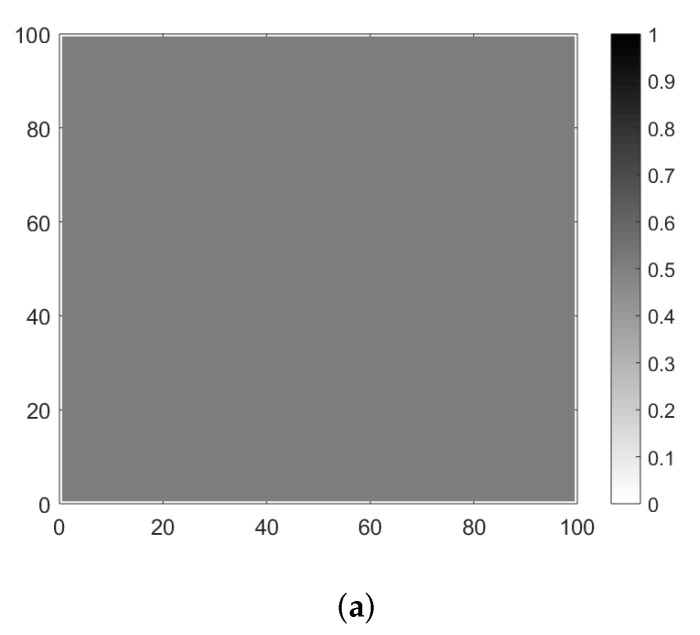

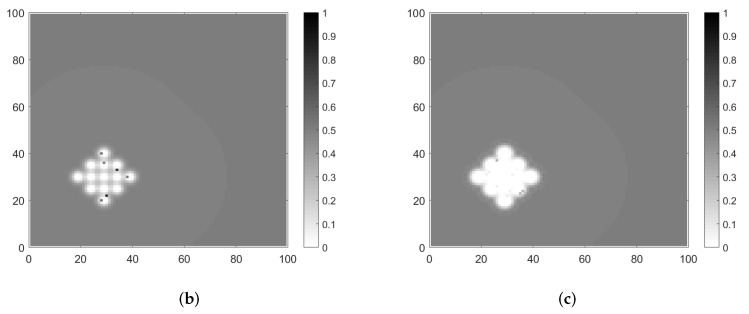
Illustration of the evolution of the POM over time: (**a**) k=0; (**b**) k=10; and (**c**) k=38. The multi-agent formation graph is shown in Figure 1a.

An illustration of the evolution of a POM and the effect of sequential Bayes update is shown in Figure 2. A group of 13 agents, in the formation shown in Figure 1a, is placed in the lower left corner of the search area of size 100×100 arbitrary units (a.u.). The target is far from all agents and cannot be detected. The sensors are characterised with parameter a=3 a.u., while the distance between the agents is 6 a.u. The probability of false alarm within the detection (sensing) volume was set to 0.005 per cell. At k=0, see Figure 2a, all pixels of the POM are set to 1/2. At k=10 (Figure 2b) and k=38 (Figure 2c), the regions of the maps in vicinity of agent locations become white, indicating a low probability of target occupancy in them. Occasional false detections increase the probability of occupancy in affected pixels; however, with time, they all tend to zero (see Figure 2c).

By staying longer in the same position, the white areas around the agent formation grow only up to a certain saturation level, determined by the probability of detection as a function of distance. The measurements received after reaching this saturation level increasingly become uninformative. For this reason, the formation at some point in time should move to another location (as discussed in the next section).

The search is terminated when the probability of occupancy in one of the cells of the POM is higher than a given threshold, i.e., when $\max_{s,n}\left\{p_{k,n}^{s}\right\}>1-\epsilon$, with ϵ≪1. The cell which satisfies this condition is declared to contain the target.

## 4. Formation Motion Control

The search objective is driven by two conflicting demands: *exploration* and *exploitation* [25]. Exploration is driving the agents to new locations in order to investigate as much of the search volume as possible. The exploitation demand is urging the agents to stay longer in one place, because this helps determine with certainty if a detection is false or true and improves the localisation accuracy. The balance between exploration and exploitation exposes two questions: how long to stay in one position and where to move next. Intermittent search strategy [13,15] was proposed as a balance between exploration and exploitation. Exploration corresponds to the ballistic flight phase, while exploitation is carried out in diffusion phase.

In decentralised multi-agent search, each agent autonomously makes a decision about its next action. However, some form of coordination between the agents is essential, in order to collectively maintain the prescribed geometric shape of the formation and thus avoid its break-up. Section 4.1 discusses the individual decisions by agents, while the team coordination is explained in Section 4.2.

### 4.1. Individual Decisions

During the diffusion phase, the formation is static and agents repeatedly collect measurements. If the sensing interval (i.e., the time required to acquire a snapshot of measurements) is $\tau_{0}$, then the duration of the diffusion phase is $\tau_{d}=m_{0}\tau_{0}$, where $m_{0}\geq 1$ is the number of sensing intervals, computed as follows. Recall that, after a single sensing interval, the probability that an agent detects a target within a circle centered at its location and with radius $L_{0}>a$, is $\exp(-L_{0}/a)$. After an arbitrary number of sensing intervals *m*, the probability that the agent *does not detect* the target within a circle of radius $L_{0}$, is then:

(4) $$P_{m}={\left[1-\exp\left(-L_{0}/a\right)\right]}^{m}$$

$P_{m}$ is monotonically decreasing with *m*. The agent should stay in one location as long as $P_{m}>p_{*}$, where $p_{*}$ is a user defined (small) probability value. Let $m=m_{0}$ be the minimal number of snapshots which satisfies $P_{m}\leq p_{*}$. After $m_{0}$ snapshots, the agent is certain with probability $1-p_{*}$, that the target is not within the radius $L_{0}$. Then from Equation (4) we can write:

(5) $$m_{0}=ceil\left[\frac{\ln(p_{*})}{\ln(1-\exp\left(-L_{0}/a\right))}\right],$$

where ceil[·] is the ceiling function (defined as the smallest integer greater than or equal to its argument).

After the diffusion phase, the agent wants to jump outside the explored area, that is, the length of its subsequent ballistic flight should be at least $L_{0}$. Let the speed of the ballistic flight be $V_{0}$. The ballistic time $\tau_{b}$, according to Bénichou et al. [13], is:

(6) $$\tau_{b}=\gamma \frac{a}{V_{0}}$$

where *a* was introduced earlier (the sensing parameter) and γ is a numerical factor dependent on the search area geometry [13]:

(7) $$\gamma ={\left[\ln\left(b/a\right)-1/2\right]}^{1/2}.$$

Note that the value of γ slowly increases with the ratio b/a. Typically, b≫a and then γ>1. The minimum length of a ballistic flight, from Equation (6) and using $\tau_{b}V_{0}=L_{0}$, equals to $L_{0}=\gamma a$. In summary, for given *a*, *b* and $p_{*}$, the count $m_{0}$ can be computed from Equation (5) as

(8) $$m_{0}=ceil\left[\frac{\ln(p_{*})}{\ln(1-\exp(-\gamma ))}\right].$$

The search starts with a diffusion phase. After collecting and exchanging all $m_{0}$ snapshot of measurements by agents in the formation, each agent compares the highest value of its local POM with a threshold, set just above 1/2, in order to test if a target have been detected. If the comparison with the threshold is positive, further investigation is required, and hence the agent moves by one step on the grid G towards the cell containing the suspected target position and repeats the diffusion phase. There are four options for this one-step move: left, right, up and down.

If the comparison is negative, the agent would consider a ballistic flight, as follows. First, it would create a set of “move” actions $U_{k}$, which consists of $|U_{k}|-1$ potential destinations for a ballistic displacement, as well as the option to remain static (no move). Let action $\alpha \in U_{k}$ represent the destination of the centroid of the formation (after the hypothetical ballistic flight). For each action $\alpha \in U_{k}$, a reward is computed.

The reward function is defined as the information gain rate, i.e.,

(9) $$R\left(\alpha \right)=\frac{H_{k}-E\left\{H_{\alpha }\right\}}{\tau_{b}+\tau_{d}}$$

where

- $H_{k}$ is the current entropy of the POM, defined as:
  (10) $$H_{k}=-\frac{1}{N}\sum_{n=1}^{N}\left[p_{k,n}\log_{2}p_{k,n}+\left(1-p_{k,n}\right)\log_{2}\left(1-p_{k,n}\right)\right]$$
- $H_{\alpha }$ is the entropy of the POM after the hypothetical move of the agent (and the entire formation) to a new destination (commanded by action α), followed by sensing and updating its local POM during the subsequent diffusion phase.
- E is the expectation operator with respect to all possible realisations of random measurements. To simplify computations, we assume that during the diffusion (sensing) phase, following an action α, sensing resulted in no detections (being the most likely scenario). In this way, we can ignore E in Equation (9).
- $\tau_{b}+\tau_{d}$ is the time required to carry out the hypothetical move and perform sensing (the ballistic time $\tau_{b}$ is the time required for the agent to travel to the new location, while $\tau_{d}$ is the sensing time in the subsequent diffusion phase). The ballistic time $\tau_{b}$ is computed as the quotient of the distance to be travelled (according to action α) and the velocity of ballistic flight $V_{0}$. Recall that one of the actions in $U_{k}$ is not to move. For this action, $\tau_{b}$ is zero.

Note that the rewards for all hypothetical actions are computed before the agent actually moves. The action which results in the maximum reward is selected and subsequently involved in the processing described in Section 4.2. Let us denote this action–reward pair for agent *s* as $\left(\alpha_{s}^{*},R_{s}^{*}\right)$.

It remains to explain how the “move” actions of $U_{k}$ are created. Their number is a parameter of the algorithm. For each “move” action, the length of the ballistic flight $\ell_{b}$ is a random draw from the exponential distribution with the mean $\bar{\ell }=\kappa L_{0}$, where κ≥1 is the multiplying factor and $L_{0}$ was introduced as the minimal length of the ballistic flight. The direction of the ballistic flight is also random: it is drawn from the uniform distribution over the interval of [0,2π] rad.

### 4.2. Coordination through Consensus

In a decentralised fusion architecture, each agent, independently of the other agents in the formation, makes a decision on its future action. This can result in a disagreement between the agents, unless the decided actions $\alpha_{s}^{*}$, s=1,…,S, are all equal. The disagreement is undesirable, because it leads to a break-up of the searching formation. The consequence of the break-up is the loss of connectivity in the communication network and, ultimately, reduced effectiveness of search. Initially, at the start of the search mission, the formation is created to ensure its communication graph is connected. The goal of cooperative control is to maintain (approximately) the shape of the formation during the mission and thereby keep the communication graph connected. In addition, it prevents a collision of agents in motion. For this reason, whenever an action decision is made by an agent, it needs to engage in a protocol which will ensure that all members of the formation reach the agreement on the common action to be applied by all of them.

The decentralised cooperative control is based on the consensus protocol [22,26]. Consensus algorithms are a family of iterative schemes which enable autonomous agents to achieve an agreement based on local information in a decentralised fusion architecture. Among the consensus protocols, the most widespread are the average consensus and the max-consensus [27]. Because we use both protocols in the proposed intermittent multi-agent search, they are briefly reviewed next.

Consider a graph representing the communication network used by the agents to share the data (such as those in Figure 1). Let S={1,2,…,S} be the set of vertices of the graph (representing the agents) and E⊆S×S the set of its edges, where an edge (s,t)∈E exists only if agents *s* and *t* can communicate directly. The set of neighbours of agent s∈S is then N(s)={t∈S:(s,t)∈E}. Suppose all vertices (agents) in the network have calculated locally a certain scalar value or “state” (such as the action reward). The goal of the max-consensus is, for each agent, to determine the globally maximum state, by communicating only with its neighbours. Let the original (initial) state of agent s∈S be denoted as $x_{s}\left(0\right)$. At each iteration τ=1,2,…, agent *s* communicates and updates its state according to [27]:

(11) $$x_{s}\left(\tau +1\right)=\max\left\{x_{s}\left(\tau \right),\max_{j\in N(s)}\left\{x_{j}\left(\tau \right)\right\}\right\}.$$

After a sufficient number of iterations (which depends on the topology of the network), all agents will reach an agreement on the global maximum.

Average consensus is an iterative algorithm by which agent s∈S computes the mean value of the collection of initial states $\left\{x_{t}\left(0\right);t=1,\ldots ,S\right\}$, by communicating only with its neighbours. At each iteration τ, agent *s* updates its state according to [22]

(12) $$x_{s}\left(\tau +1\right)=q_{ss}x_{s}\left(\tau \right)+\sum_{j\in N(s)}q_{sj}x_{j}\left(\tau \right)$$

where $Q=[q_{ij}]$ is referred to as the averaging matrix. *Q* must be symmetric and doubly stochastic [28], and satisfies $q_{ij}=0$ if (i,j)∉E. We adopt the averaging matrix as the Metropolis weight matrix [29]:

(13) $$q_{ij}=\left\{\begin{array}{cc} \frac{1}{1+\max\{|N(i)|,|N(j)|\}} & \mathrm{if}\;(i,j)\in E\\ 1-\sum_{(i,t)\in E}q_{it} & \mathrm{if}\;i=j\\ 0 & \mathrm{otherwise} \end{array}\right.$$

The consensus algorithm is iterative and hence its convergence properties are very important. Although the network topology may change with time (as the agents move), during the short interval, when the exchange of information takes place, the topology can be considered as time-invariant. The convergence of the consensus algorithm in the adopted framework (time-invariant, undirected communication topology) is guaranteed, if the graph is connected [22,29,30]. While this is a theoretical result, valid for an infinite number of iterations, in practice, due to the finite number of iterations, the consensus may not be reached and, as a consequence, one or more agents may be lost during the search (a lost agent has lost the connection with the formation). This event, however, does not mean that the search mission has failed: the target can be found eventually by the remaining (smaller) formation, albeit in a (possibly) longer interval of time.

The max-consensus is used by agents to agree on: (1) the destination of the ballistic flight with the overall highest reward; and (2) the termination of search. The average consensus is used to agree with the simple majority in which direction (left, right, up or down) to carry out the one-step move towards the closest potential target.

## 5. Numerical Results

### 5.1. An Illustrative Run

**Figure 3:**
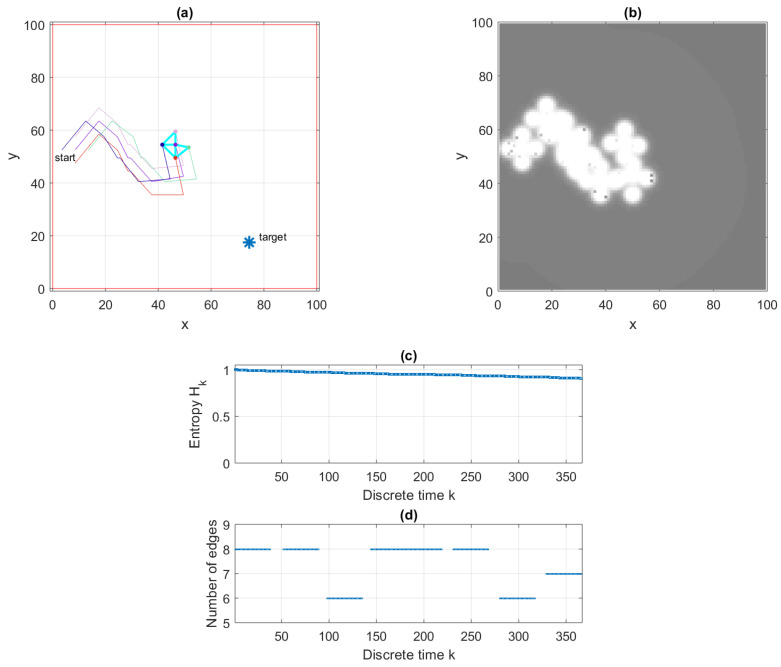
An illustrative run with S=5 agents: (**a**) a top-down view of the search area and agents’ paths up to k=375; (**b**) the estimated POM of agent number 1 at k=375 (the shades of gray indicate the value of probability); (**c**) entropy $H_{k}$ as a function of time; and (**d**) cardinality of the set $E_{k}$ as a function of time.

The simulation setup is shown in Figure 3a. The search area is a square of size b=100 a.u. A target is placed at coordinates (19,75) a.u. The searching formation consists of five agents, which initially have a shape of a square of size d=5, with $R_{\max}=1.5\;d$. Figure 3a shows the paths of the five searching agents from k=1 to k=375. The cyan-coloured lines connecting the agents at k=375 indicate the established communication links (i.e., the edges of the communication graph). The parameters used in this illustrative run were a=1 and $p_{*}=0.005$, which results in $m_{0}=37$. Furthermore, κ=3, $V_{0}=1$ and ϵ=0.0025. Each agent suggests nine ballistic flight destinations (i.e., the cardinality $|U_{k}|=10$). The number of iterations of the consensus algorithm, both for decentralised estimation and decentralised control, was fixed to 30.

Figure 3b displays the estimated POM of agent number 1 at k=375. The white areas (traversed and examined by the formation) indicate a low probability of target presence. Occasional black dots in the white regions are due to false detections. The entropy $H_{k}$ of the POM as a function if time is shown in Figure 3c. Finally, the number of established communication links in the network, as a function of time, is displayed in Figure 3d. The gaps in this figure correspond to the ballistic flight intervals. A video of a single run of the algorithm can be found in the Supplementary Materials.

### 5.2. Monte Carlo Runs

**Figure 4:**
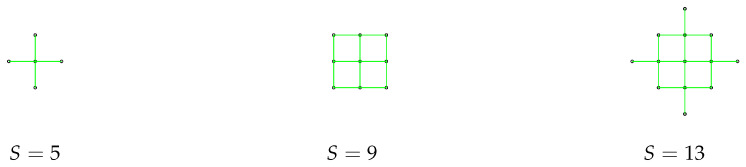
Multi-agent formations (and their initial communication graphs) used in Monte Carlo simulations. The minimum distance d=5, $R_{\max}=1.4\;d$.

Monte Carlo runs were performed to compare the performance of four different searching formations: a single agent (S=1) and formations with S=5,9 and 13 agents. The initial communication graph of multi-agent formations are shown in Figure 4. The minimum distance between the agents is d=5, with $R_{\max}=1.4\;d$. The search area is a square of size b=100 a.u. The parameters were: a=1, $p_{*}=0.005$, κ=3, $V_{0}=1$ and ϵ=0.0025. Each agent suggests nine ballistic flight destinations.

**Figure 5:**
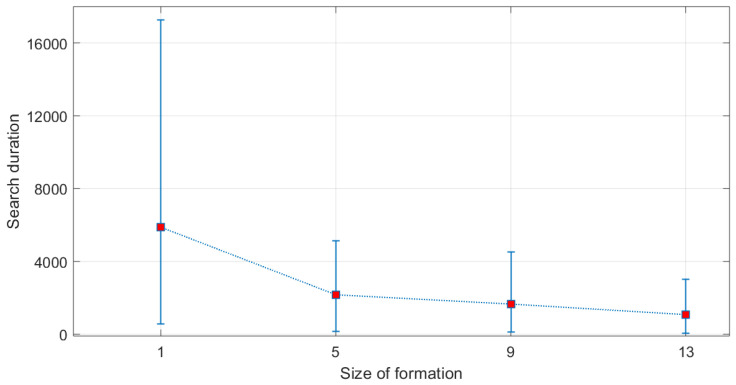
Search time duration as a function of the size of formation *S*: the mean (red squares) and the [5,95] percentile limits.

The number of Monte Carlo runs was set to 100. The obtained average search duration is shown in Figure 5, including the 5th and 95th percentile limits. The average search duration is also given in the second row of Table 1. In accordance with our expectations, the larger is the formation, the shorter is the search time. The law of diminishing returns is also evident: the benefit (i.e., the search time reduction) is slowly reducing with *S*. Other average statistics recorded from Monte Carlo runs are shown in Table 1, in particular: (i) the average (over time and Monte Carlo runs) number of edges in the communication graph (third row); (ii) the average (over time and Monte Carlo runs) number of lost agents (fourth row); and (iii) the fraction of Monte Carlo runs in which the target was successfully located. The only surprising statistic is that the mean number of lost agents is smaller for the formation with S=9 than for the other two. This is probably due to the shape of this formation, in which each agent is connected to at least two other nodes (see Figure 4).

Next, we discuss the alternative search strategies. One option is a pure diffusion phase of search, where the agents move at most by one step on the grid and sense. This type of search would naturally take much longer to find the target, possibly an infinite length of time. Another option is the intermittent search, where the choice of the ballistic flight is purely random (instead of using the ballistic flight with a maximum reward (Equation (9))). The mean search time with S=5 agents (all other parameters the same as above) was 4389 a.u using a random choice versus 2250 a.u. using the information reward. The benefit of the information reward is evident.

## 6. Conclusions

This report presents an intermittent information-driven search strategy by a team of coordinated agents. Both the estimation of the target occupancy map and the motion control for the entire formation were carried out in a decentralised manner, meaning that all computations were done locally. Coordination of agents was achieved using the consensus algorithm, which is an iterative scheme in which the data are exchanged with neighbours only, in a manner which does not require the global knowledge of the communication network size and topology. Numerical results indicate a high success rate in finding the target with search duration inversely proportional to the size of the multi-agent formation.

Future work could incorporate more complex motion of the searching formation, including its rotation and scaling (growing and contracting). This has a potential to further reduce the search time, especially for larger formations.

## Figures and Tables

**Table 1:** Multi-agent search statistics.

*S*	1	5	9	13
Means search duration (a.u.)	5134	2250	1329	857
Mean |E|	-	4.37	12.79	17.88
Mean Lost	-	0.36	0.13	0.30
Success	0.93	0.95	0.95	0.95

## Supplementary Materials

The following are available online at https://zenodo.org/record/1248482#.XvmbmOcRVPZ.

## References

[1] Ristic B., Skvortsov A., Gunatilaka A. (2016). A study of cognitive strategies for an autonomous search. Inf. Fusion.

[2] Hutchinson M., Liu C., Chen W.H. (2018). Information-Based Search for an Atmospheric Release Using a Mobile Robot: Algorithm and Experiments. IEEE Trans. Control. Syst. Technol..

[3] Haley K.B., Stone L.D. (1980). Search Theory and Applications (Nato Conference Series).

[4] Stone L.D. In search of Air France flight 447. Institute of Operations Research and the Management Sciences 2011.

[5] Halford S.E. (2004). How do site-specific DNA-binding proteins find their targets?. Nucleic Acids Res..

[6] Viswanathan G.M., Afanasyev V., Buldyrev S.V., Murphy E.J., Prince P.A., Satnley H.E. (1996). Levy fligh search patterns of wandering albatrosses. Nature.

[7] Fauchald P., Tveraa T. (2003). Using first-passage time in the analysis of area-restricted search and habitat selection. Ecology.

[8] Chung T.H., Hollinger G.A., Isler V. (2011). Search and pursuit-evasion in mobile robotics. Auton. Robot..

[9] Koopman B.O. (1946). Search and screening.

[10] Champagne L., Carl E.G., Hill R. Search theory, agent-based simulation, and u-boats in the Bay of Biscay. Proceedings of the 2003 Winter Simulation Conference.

[11] Bernardini S., Fox M., Long D. (2017). Combining temporal planning with probabilistic reasoning for autonomous surveillance missions. Auton. Robot..

[12] Shlesinger M.F. (2006). Mathematical physics: Search research. Nature.

[13] Bénichou O., Loverdo C., Moreau M., Voituriez R. (2011). Intermittent search strategies. Rev. Mod. Phys..

[14] Kramer D.L., McLaughlin R.L. (2001). The Behavioral Ecology of Intermittent Locomotion. Am. Zool..

[15] Bénichou O., Loverdo C., Moreau M., Voituriez R. (2006). Two-dimensional intermittent search processes: An alternative to Lévy flight strategies. Phys. Rev. E.

[16] Vergassola M., Villermaux E., Shraiman B.I. (2007). ‘Infotaxis’ as a strategy for searching without gradients. Nature.

[17] Masson J.B., Bailly-Bachet M., Vergassola M. (2009). Chasing information to search in random environments. J. Phys. A Math. Theor..

[18] Hajieghrary H., Hsieh M.A., Schwartz I.B. (2016). Multi-agent search for source localization in a turbulent medium. Phys. Lett. A.

[19] Ristic B., Gilliam C., Moran W., Palmer J.L. (2020). Decentralised multi-platform search for a hazardous source in a turbulent flow. Inf. Fusion.

[20] Park M., Oh H. (2020). Cooperative information-driven source search and estimation for multiple agents. Inf. Fusion.

[21] Song C., He Y., Ristic B., Lei X. (2020). Collaborative infotaxis: Searching for a signal-emitting source based on particle filter and Gaussian fitting. Robot. Auton. Syst..

[22] Olfati-Saber R., Fax J.A., Murray R.M. (2007). Consensus and cooperation in networked multi-agent systems. Proc. IEEE.

[23] Krout D.W., Fox W.L.J., El-Sharkawi M.A. (2009). Probability of target presence for multistatic sonar ping sequencing. IEEE J. Ocean. Eng..

[24] Hlinka O., Hlawatsch F., Djuric P.M. (2013). Distributed particle filtering in agent networks: A survey, classification, and comparison. IEEE Signal Process. Mag..

[25] Cohen J.D., McClure S.M., Angela J.Y. (2007). Should I stay or should I go? How the human brain manages the trade-off between exploitation and exploration. Philos. Trans. R. Soc. B.

[26] Ren W., Beard R., Atkins E. (2007). Collective Group Behavior Through Local Interaction. IEEE Control. Syst..

[27] Nejad B.M., Attia S.A., Raisch J. (2010). Max-consensus in a max-plus algebraic setting: The case of switching communication topologies. IFAC Proc. Vol..

[28] Dimakis A.G., Kar S., Moura J., Rabbat M.G., Scaglione A. (2010). Gossip algorithms for distributed signal processing. Proc. IEEE.

[29] Xiao L., Boyd S., Lall S. A scheme for robust distributed sensor fusion based on average consensus. Proceedings of the IPSN 2005 Fourth International Symposium on Information Processing in Sensor Networks.

[30] Ren W., Beard R.W., Atkins E.M. (2007). Information consensus in multivehicle cooperative control. IEEE Control. Syst. Mag..

